# Sniffing out Stingray Noses: The Functional Morphology of Batoid Olfaction

**DOI:** 10.1093/iob/obac043

**Published:** 2022-10-10

**Authors:** K M Rutledge

**Affiliations:** Department of Ecology and Evolutionary Biology, University of California Los Angeles, Los Angeles, California 90095, USA

## Abstract

Batoid fishes (rays, skates, sawfishes, and guitarfishes) are macrosmatic, meaning they rely on their sense of smell as one of the primary senses for survival and reproduction. Olfaction is important for long-distance tracking and navigation, predator and prey recognition, and conspecific signaling. However, the mechanisms by which batoids harness odorants is unknown. Without a direct pump-like system, it is hypothesized that batoids irrigate their nostrils via one or a combination of the following: the motion pump, buccopharyngeal pump, pressure (ex. pitot-like mechanism), or a shearing force (ex. viscous entrainment). These mechanisms rely on the size, shape, and position of the nostrils with respect to the head and to each other. Batoids are united as a group by their dorsoventrally compressed body plans, with nostrils on the ventral side of their body. This position presents several challenges for odor capture and likely limits the effectivity of the motion pump. Batoid fishes display an expansive nasal morphology, with inlet nostrils ranging from thin, vertical slits to wide, horizontal ovals to protruding, tube-like funnels, and more. In this paper, a morphometric model is developed to quantify the vast diversity in batoid nose shapes, sizes, and positions on the head in an ecological and functional framework. Specifically, swimming mode, lifestyle, habitat, and diet are examined for correlations with observed nasal morphotypes. Morphometric measurements were taken on all 4 orders present in Batoidea to broadly encompass batoid nasal diversity (Rhinopristiformes 4/5 families; Rajiformes 2/4 families; Torpediniformes 4/4 families; Myliobatiformes 8/11 families). All batoid external nasal diversity was found to be categorized into 5 major morphological groups and were termed: flush nare [circle, comma, intermediate], open nare, and protruding nare. Several morphometric traits remained significant when accounting for shared ancestry, including the position and angle of the nostril on the head, the width of the inlet hole, and the spacing of the nostrils from each other. These measurements were found to be closely correlated and statistically significant with the swimming mode of the animal. This study provides the first crucial step in understanding batoid olfaction, by understanding the diversity of the morphology of the system. Because odor capture is a strictly hydrodynamic process, it may be that factors relating more directly to the fluid dynamics (i.e., swimming mode, velocity, Reynolds number) may be more important in shaping the evolution of the diversity of batoid noses than other ecological factors like habitat and diet.

## Spanish

Los peces batoideos (rayas, rayas, peces sierra y peces guitarra) son macrosmáticos, lo que significa que dependen de su sentido del olfato como uno de los sentidos principales para la supervivencia y la reproducción. El olfato es importante para el seguimiento y la navegación a larga distancia, el reconocimiento de depredadores y presas y la señalización conespecífica. Sin embargo, se desconocen los mecanismos por los cuales los batoideos aprovechan los olores. Sin un sistema similar a una bomba directa, se plantea la hipótesis de que los batoideos irrigan sus fosas nasales a través de uno o una combinación de los siguientes: la bomba de movimiento, la bomba bucofaríngea, la presión (por ejemplo, un mecanismo tipo pitot) o una fuerza de corte (por ejemplo, viscosa). arrastre). Estos mecanismos se basan en el tamaño, la forma y la posición de las fosas nasales con respecto a la cabeza y entre sí. Los batoideos están unidos como grupo por sus planes corporales comprimidos dorsoventralmente, con fosas nasales en el lado ventral de su cuerpo. Esta posición presenta varios desafíos para la captura de olores y probablemente limita la efectividad de la bomba de movimiento. Los peces batoideos muestran una morfología nasal expansiva, con fosas nasales de entrada que van desde hendiduras verticales delgadas hasta óvalos horizontales anchos, embudos que sobresalen en forma de tubo y más. En este artículo, se desarrolla un modelo morfométrico para cuantificar la gran diversidad de formas, tamaños y posiciones de la nariz de los batoides en la cabeza en un marco ecológico y funcional. Específicamente, el modo de natación, el estilo de vida, el hábitat y la dieta se examinan en busca de correlaciones con los morfotipos nasales observados. Se tomaron medidas morfométricas en los 4 órdenes presentes en Batoidea para abarcar ampliamente la diversidad nasal de batoides (Rhinopristiformes 4/5 familias; Rajiformes 2/4 familias; Torpediniformes 4/4 familias; Myliobatiformes 8/11 familias). Se encontró que toda la diversidad nasal externa de los batoides se clasificaba en 5 grupos morfológicos principales y se denominaba: nariz rasante [círculo, coma, intermedio], nariz abierta y nariz sobresaliente. Varios rasgos morfométricos siguieron siendo significativos al tener en cuenta la ascendencia compartida, incluida la posición y el ángulo de la fosa nasal en la cabeza, el ancho del orificio de entrada y el espacio entre las fosas nasales. Se encontró que estas medidas estaban estrechamente correlacionadas y eran estadísticamente significativas con el modo de natación del animal. Este estudio proporciona el primer paso crucial para comprender el olfato de los batoides, al comprender la diversidad de la morfología del sistema. Debido a que la captura de olores es un proceso estrictamente hidrodinámico, es posible que los factores que se relacionan más directamente con la dinámica de fluidos (es decir, el modo de natación, la velocidad, el número de Reynolds) sean más importantes para dar forma a la evolución de la diversidad de narices de batoides que otros factores ecológicos como hábitat y dieta.

## German

Fledermausfische (Rochen, Rochen, Sägefische und Gitarrenfische) sind makrosmatisch, was bedeutet, dass sie sich auf ihren Geruchssinn als einen der wichtigsten Sinne für Überleben und Fortpflanzung verlassen. Der Geruchssinn ist wichtig für die Verfolgung und Navigation über große Entfernungen, die Erkennung von Raubtieren und Beute sowie die Signalgebung von Artgenossen. Die Mechanismen, mit denen Batoide Geruchsstoffe nutzen, sind jedoch unbekannt. Ohne ein direktes pumpenähnliches System wird angenommen, dass Batoide ihre Nasenlöcher über eines oder eine Kombination der folgenden spülen: die Bewegungspumpe, die bukkopharyngeale Pumpe, den Druck (z. B. Pitot-ähnlicher Mechanismus) oder eine Scherkraft (z. B. viskos Mitnahme). Diese Mechanismen beruhen auf der Größe, Form und Position der Nasenlöcher in Bezug auf den Kopf und zueinander. Batoide werden durch ihre dorsoventral komprimierten Körperpläne als Gruppe vereint, mit Nasenlöchern auf der ventralen Seite ihres Körpers. Diese Position stellt mehrere Herausforderungen für die Geruchserfassung dar und schränkt wahrscheinlich die Wirksamkeit der Bewegungspumpe ein. Batoid-Fische weisen eine ausgedehnte Nasenmorphologie auf, mit Einlassnasen, die von dünnen, vertikalen Schlitzen über breite, horizontale Ovale bis hin zu hervorstehenden, röhrenartigen Trichtern und mehr reichen. In diesem Artikel wird ein morphometrisches Modell entwickelt, um die große Vielfalt an Batoid-Nasenformen, -größen und -positionen auf dem Kopf in einem ökologischen und funktionalen Rahmen zu quantifizieren. Insbesondere werden Schwimmmodus, Lebensstil, Lebensraum und Ernährung auf Korrelationen mit beobachteten nasalen Morphotypen untersucht. Morphometrische Messungen wurden an allen 4 in Batoidea vorkommenden Ordnungen durchgeführt, um die Diversität der Batoidnasen breit zu erfassen (Rhinopristiformes 4/5 Familien; Rajiformes 2/4 Familien; Torpediniformes 4/4 Familien; Myliobatiformes 8/11 Familien). Es wurde festgestellt, dass die gesamte Vielfalt der batoiden äußeren Nase in 5 morphologische Hauptgruppen eingeteilt und als bündige Nasenöffnung [Kreis, Komma, Zwischen], offene Nasenöffnung und hervorstehende Nasenöffnung bezeichnet wurde. Mehrere morphometrische Merkmale blieben bei der Berücksichtigung der gemeinsamen Abstammung signifikant, einschließlich der Position und des Winkels des Nasenlochs am Kopf, der Breite des Einlasslochs und des Abstands der Nasenlöcher voneinander. Es wurde festgestellt, dass diese Messungen eng mit dem Schwimmmodus des Tieres korrelieren und statistisch signifikant sind. Diese Studie liefert den ersten entscheidenden Schritt zum Verständnis des batoiden Geruchssinns, indem sie die Vielfalt der Morphologie des Systems versteht. Da die Geruchsbindung ein streng hydrodynamischer Prozess ist, kann es sein, dass Faktoren, die sich direkter auf die Fluiddynamik beziehen (z. B. Schwimmmodus, Geschwindigkeit, Reynolds-Zahl), wichtiger für die Gestaltung der Entwicklung der Vielfalt von Fledermausnasen sind als andere ökologische Faktoren wie z Lebensraum und Ernährung.

## Introduction

A successful chemical detection system must have both accurate and precise internal sensors and a reliable odor harnessing and directing system. For a fish to detect an odorant, a chemical signal must traverse the external fluid environment, funnel into the nose, and bind with an odorant receptor. The odorant receptor must identify the specific odorant and send a signal to the brain with information on the nature of the chemical signal. Thus, a fish's olfactory system can be generally broken into two major components: (1) the external interaction with the environment and (2) the internal flow and sensory mechanics. While there have been numerous studies looking at the internal sensory architecture of a fish's nose (with an impressive sub-nanomolar detection capability; Meredith and Kaijura 2010), there is far less research on the external morphology and odor harnessing mechanisms.

Batoid fishes (rays, skates, sawfishes, and guitarfishes) present a unique system to study the morphology and fluid dynamics of olfaction for several reasons. First, batoid fishes have an expansive diversity in external nasal morphology, including differences in nostril shapes, sizes, relative positions, and external protruding features. Second, they are dorsoventrally compressed, with their nostrils positioned ventrally and medial on the underside of their body near their mouth and gills. Third, batoid fishes are macrosmatic, meaning they rely on their sense of smell as one of their primary senses for survival and reproduction (Collins et al. 2015). This includes long-distance tracking and navigation, predator and prey recognition, and conspecific signaling (Gardiner et al. 2012, 2014; Hart and Collin 2015). Fourth, the niche space of batoids is expansive, with diversification into many environments and life histories. Finally, they lack an apparent pump-like mechanism to irrigate their nostrils. Their nostrils are disconnected from the pharynx and mouth, and they lack olfactory accessory sacs, the water-pumping nasal chambers seen in many teleost fishes. Without a direct pump-like mechanism and this ventral positioning, how are odorants efficiently captured? With this morphological nasal diversity in mind, are there unifying properties across species? How do these design parameters change with diversification into different habitats and lifestyles?

**Fig. 1:**
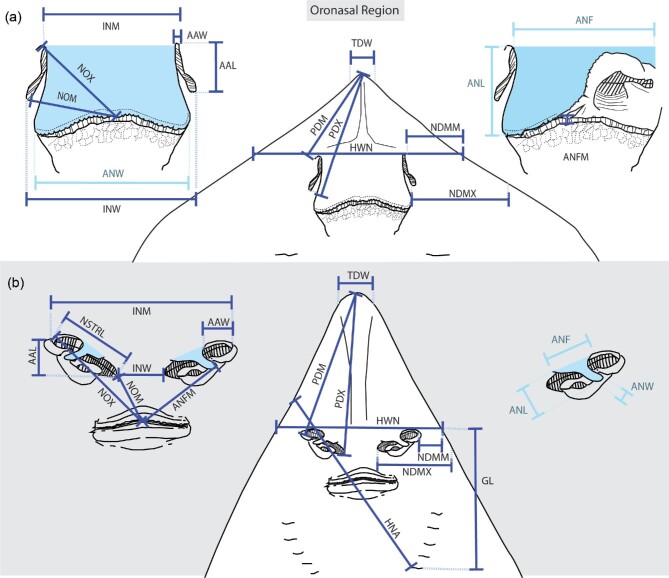
The morphometric measurements used in this study to describe batoid nose morphology. Measurements are outlined on two disparate morphotypes, as shown on a generic (a) urotrygonid stingray and (b) rhinobatid guitarfish. The central drawings show the ventral view of the head and oronasal region. The flanking drawings are of the nose and mouth (oronasal region) isolated from the head. The incurrent channel is designated by the anterior aperture width (AAW). The nasal curtain in stingrays and anterior nasal flap in guitarfish (ANF), a homologous structure, is colored in light blue. The excurrent channel in stingrays is underneath the lifted nasal curtain, shown on the right-hand side drawing. The excurrent channel in guitarfishes is to the left of the anterior nasal flap.

To answer these functional questions, the morphology of the system must first be examined. The typical arrangement of the olfactory organ of batoids is found as a blind chamber partially divided by an anterior, inlet nostril (termed the incurrent nostril) though which water enters and a posterior, outlet channel (excurrent nostril) through which water leaves (Tester 1963; Theisen et al. 1986; Zeiske et al. 1987; Compagno 1999; Abel et al. 2010). The incurrent nostrils (also called the nares) range from thin, vertical slits to wide, horizontal ovals to protruding tube-like funnels, and more. Batoids also possess one or more dividing nasal flaps flanking the incurrent nostrils. Many batoids have one major nasal flap, called the nasal curtain, which is an elongated flap of tissue that extends from the medial side of the incurrent nostrils to the anterior edge of the mouth (Fig. 1a). This curtain loosely covers the excurrent channel of the nose and forms the respective outlet hole. This curtain is also variable in shape, size, and length with respect to the mouth. In addition to the nasal curtain, some species possess additional flaps of tissue that flank or project from the incurrent nostril hole. In species that lack a nasal curtain (guitarfishes and sawfishes), these nasal flaps act as rudimentary divisions between the nasal chamber, designating the inlet from the outlet (Fig. 1b). Housed inside the nostril is the olfactory rosette, which is composed of a longitudinal array of numerous (up to 300) flexible, parallel plates of tissue, called lamellae. The lamellae have numerous, microscopic folds called secondary folds that increase the surface area of the structure (Ferrando et al. 2017). The lamellae are coated in non-sensory and sensory epithelium (Takami et al. 1994; Meredith and Kajiura 2010; Ferrando et al. 2017; Simonitis and Marshall 2022). The sensory epithelium is coated in supporting cilia and houses the olfactory receptor neurons (Theisen et al. 1986; Zeiske et al. 1987; Schluessel et al. 2008). It is unknown whether the supporting cilia propels mucus or water, but evidence suggests they likely propel mucus (Cox 2013). To receive olfactory information for sensory processing, odorants must enter the inlet nostril, pass through the incurrent channel and across the olfactory rosette where it then binds with an olfactory receptor neuron on the lamellae that sends a signal to the brain via the olfactory bulb (Yopak et al. 2015).

Therefore, the shape, size, and position of a fish's nostrils determine how odor is captured and transported (Settles 2005; Cox 2008). Fishes will orient into an odor plume by comparing the bilateral odor concentration differences of their paired nostrils, turning towards the higher concentration, or the nostril that is stimulated first (Tester 1963; Bardach Todd, and Crickmer, 1967; Atema 1971; Mathewson and Hodgson 1972; Johnson and Teeter 1985; Gardiner and Atema, 2010). Therefore, the spacing and position of the paired nostrils will directly impact its olfactory abilities. Additionally, because many fishes, including batoids, lack a direct pump-like system to irrigate their nostrils, they must rely on harnessing external flows to capture odorants. These include the relative forward motion of a swimming fish (motion-pump), indirect respiratory flow (buccopharyngeal pump), pitot- and venturi-like mechanisms, and viscous entrainment (Vogel 1977). These hypothesized ventilation methods rely on nare morphology and position, with incurrent and excurrent nostrils positioned at right angles (pitot), different heights (venturi), or perpendicular to flow (viscous entrainment) allowing for the generation of a secondary flow through pressure differences or a shearing force (Cox 2008). Most bony fishes have small, circular nostrils located on the most anterior, dorsal part of their head. At this anterodorsal position, a fish facing into a current will capture odorants by funneling them into the forward-facing incurrent nostrils through the motion-pump (Vogel 1977; Garwood et al. 2019, 2020). This position, almost on the apex of the snout, also minimizes the odor-impeding effects of the boundary layer (the stationary layer of fluid surrounding a swimming fish) (Cox 2008). However, the nostrils of batoid fishes, on the underside of their body, are positioned in a thicker region of the boundary layer and not in the direct path of water flow, likely limiting the effectivity of the motion pump. This may have influenced the expansive external nasal morphology observed in batoids. This unique positioning and disparate morphology will impact the way batoids sense and harness odorants.

The hypothesized odor-harnessing mechanisms in batoids may also depend on environmental and species-specific factors. Possible relevant ecological influences include swimming mode, position in the water column (benthic vs. pelagic), habitat, and diet. Batoids exhibit a variety of swimming modes, including oscillatory, undulatory, and body caudal fin swimming. Many benthic species also display a form of locomotion called “punting,” where the animal is in close association with the ground. Each of these swimming modes will influence the pitch, yaw, and swimming speed of the animal. It is likely that for these reasons, swimming mode will directly influence how odorants are tracked and ultimately harnessed into the nose. The position in the water column will also likely influence odor capture, with pelagic species known to rely on the motion-pump as they swim at fast speeds (Cox 2008). With fewer sensory cues in the open ocean, pelagic species may also rely more on olfaction than other ecologies. In fact, pelagic batoids were found to possess significantly more olfactory lamellae and larger sensory epithelial surface area than benthic species (Schluessel et al. 2008). Benthic species are generally slower swimmers and odor capture is likely affected by the proximity to the substrate and the associated ground effects. While it has not been explored in batoids, benthic sharks were found to have more complex nasal morphologies, with multiple flap-like structures that may aid in odor uptake. The nostrils of benthic sharks are also positioned closer to their mouth, suggesting the indirect respiratory current may aid in nasal irrigation (Timm and Fish 2012). Similar to batoids, shark nostrils are also considered ventrally positioned, but unlike batoids their incurrent nostrils are anterior and forward-facing in the transverse plane of their body, allowing for a more optimal configuration for harnessing odorants via the motion pump.

Habitat may have also influenced the evolution of batoid nasal morphology (Hara 1993; Kajiura 2001; Schluessel et al. 2008; Timm and Fish 2012). Batoids living in the deep sea or murky waters were found to have larger olfactory bulbs, a region of the brain that is used as a reliable proxy for olfactory capability (Lisney and Collin 2007; Theiss et al. 2009; Yopak et al. 2015; Camilieri-Asch et al. 2020). Reef-associated species were found to possess the smallest olfactory bulbs, possibly suggesting reliance on other senses such as vision or electroreception (Hart et al. 2006; Collin 2012; Kempster et al. 2012; Lisney et al., 2012; Yopak et al. 2015). Additionally, batoids that rely heavily on olfactory cues for spatial navigation, such as long-distance migration or active prey tracking and localization, may have a more acute olfactory system (Jacobs 2012). This has been unexplored in batoids, but the highly migratory Port Jackson shark was found to have an especially large sensory surface area, olfactory rosette mass, and deep secondary folding on the lamellae, suggesting an increased olfactory ability (Timm and Fish 2012). While certain ecologies appear to have increased olfactory abilities, it is unknown if these ecologies have also evolved specific odor harnessing morphological adaptations.

While ecological trends in internal nasal morphology (lamellae count, surface area) and brain size (olfactory bulb volume) have been explored in batoids, the external morphology has received far less attention. Specifically, there are no studies that have quantified the external nasal morphology of batoids in relation to their ecology. This study broadly classifies external batoid nasal diversity in a quantitative, ecological, and phylogenetic framework. Specifically, a morphometric model is created for quantifying the observed diversity in batoid morphology into discrete morphotypes. Relevant ecological parameters (swimming mode, lifestyle, habitat, and diet) are examined for correlations with observed nasal morphotypes. I hypothesized that the diversity seen in batoid nasal morphology is more than just the result of shared ancestry, with convergence on certain morphotypes that may act as functional adaptations for odor capture. This study lays the foundation for better understanding the evolution and function of a unique olfactory system that likely relies heavily on its morphology to capture and direct odorants.

## Methods

### Morphometric Model

A morphometric model was created with 18 measurements to broadly encompass nose shape, size, angle, and position on the head across batoids. Measurements included and were designated as: prenarial distance minimum (PDM), prenarial distance maximum (PDX), tip of nose width (TDW), head width at narial opening (HWN), narial oral distance minimum (NOM), narial oral distance maximum (NOX), distance from nostril to disc margin minimum (NDMM), distance from nostril to disc margin maximum (NDMX), head nostril angle (HNA), anterior nasal flap to mouth distance (ANFM), nostril length (NOW), anterior aperture width/nare opening (AAW), exposed nostril length (NSTRL), distance across anterior nasal apertures (INM), internarial distance minimum(INW), anterior nasal flap base length (ANF), anterior nasal flap length (ANL), anterior nasal flap distal width (ANW), proximal angle of nare (PAN), and head narial angle (HNA). Exact measurement specifications are diagrammed in Fig. 1 on two opposing nasal morphotypes. Acronyms mostly follow terminology in Last et. al 2016b, however, some are newly created here and some acronyms were changed for clarity (ex. ANW is anterior nasal flap width here, but anterior nasal flap length in Last et al. 2016b). Nasal curtain (anterior nasal flap) terminology also follows Last et al. 2016b, including: square (the anterior and posterior width of the nasal curtain are approximately the same), skirt-shaped (the anterior width is narrower than the posterior width, forming a “skirt” or triangular shape), incomplete (the posterior edge of the nasal curtain is incompletely joined, forming two lobes that make a “W” shape), and reduced (the nasal curtain is reduced to a small flap that does not cover the nasal channel or outlet).

### Measurements and replicates

Eighteen different morphometric measurements relating to nose morphology were taken on over 144 adult individuals (Fig. 1). These data span all 4 orders present in Batoidea, 17 families and 28 genera, to broadly encompass batoid nasal diversity (Rhinopristiformes 4/5 families; Rajiformes 2/4 families; Torpediniformes 4/4 families; Myliobatiformes 8/11 families) (Table 1; Supplementary Table 1). The purpose of taxon selection was to sample broadly across the phylogeny to capture as much variation in nose morphology as possible. There were no significant differences in external nose morphology between species in the same genus therefore sampling was focused on maximizing genera. In instances where replicates of the same species were rare in visited collections (ex. *Mobula*) other species within the same genus were considered replicates for that genus. There was also no morphological sexual dimorphism in external nasal morphology, which agrees with the same conclusion of previous studies on internal nasal morphology (Schluessel et al. 2010). Preliminary analysis for determining the appropriate sample size found that there was no difference in standard error between 3, 6, and 11 replicate individuals in the genus *Pseudobatos* and *Myliobatis*. Replicates ranged from 2–11 individuals per species, with an average of 5 individuals. However, there were 2 species (*Pteroplatytrygon violacea* and *Rhina ancylostoma)* where the minimum sample size of 3 was not possible due to rarity in visited collections.

**Table 1:** Representative species in this study across all four orders in Batoidea and 17 families classified by nasal curtain and the newly described here “nare type.”

Order	Family	Species	Nasal curtain	Nare type	Replicates
Rajiformes	Arhynchobatidae	*Bathyraja kincaidii*	incomplete	protruding	6
Rajiformes	Arhynchobatidae	*Psammobatis scobina*	incomplete	protruding	4
Rajiformes	Rajidae	*Beringaja rhina*	incomplete	protruding	5
Myliobatiformes	Aetobatidae	*Aetobatus narinari*	incomplete	comma	6
Myliobatiformes	Dasyatidae	*Taeniura lymma*	skirt shaped	intermediate	4
Myliobatiformes	Dasyatidae	*Neotrygon kuhlii*	skirt shaped	intermediate	4
Myliobatiformes	Dasyatidae	*Pteroplatytrygon violacea*	skirt shaped	comma	2
Myliobatiformes	Dasyatidae	*Hypanus longus*	square	comma	7
Myliobatiformes	Dasyatidae	*Himantura uarnak*	skirt shaped	comma	4
Myliobatiformes	Gymnuridae	*Gymnura crebripunctata*	skirt shaped	circle	10
Myliobatiformes	Hypnidae	*Hypnos monopterygius*	incomplete	circle	3
Myliobatiformes	Mobulidae	*Mobula hypostoma*	square	circle	2
Myliobatiformes	Myliobatidae	*Aetomylaeus nichofii*	square	intermediate	4
Myliobatiformes	Myliobatidae	*Myliobatis californica*	square	intermediate	11
Myliobatiformes	Rhinopteridae	*Rhinoptera bonasus*	skirt shaped	intermediate	5
Myliobatiformes	Urotrygonidae	*Urotrygon aspidura*	skirt shaped	comma	4
Myliobatiformes	Urotrygonidae	*Urobatis maculatus*	skirt shaped	comma	7
Myliobatiformes	Potamotrygonidae	*Potamotrygon orbignyi*	square	intermediate	6
Rhinopristiformes	Rhinidae	*Rhina ancylostoma*	reduced	open	2
Rhinopristiformes	Rhinobatidae	*Trygonorrhina fasciata*	reduced	intermediate	3
Rhinopristiformes	Rhinobatidae	*Zapteryx exasperata*	reduced	open	6
Rhinopristiformes	Rhinobatidae	*Glaucostegus granulatus*	reduced	open	4
Rhinopristiformes	Rhinobatidae	*Pseudobatos leucorhynchus*	reduced	open	11
Rhinopristiformes	Trygonorrhinidae	*Platyrhinoidis triseriata*	reduced	open	5
Torpediniformes	Torpedinidae	*Tetronarce californica*	square	protruding	5
Torpediniformes	Narcinidae	*Benthobatis yangi*	square	circle	5
Torpediniformes	Narcinidae	*Narcine entemedor*	square	circle	5
Torpediniformes	Narkidae	*Narke japonica*	square	protruding	3

### Choosing the representative specimen

One replicate specimen was chosen to be the representative species for each genus. The goal of this method was to minimize distortion of these data that can come with averaging replicates. Instead of analyzing data from a hypothetical species that was the result of averaged measurements, I analyzed data from the actual measurements from the most representative specimen (Burns and Sidlauskas 2019). To choose which replicate would be the representative, I used PCA plots to find the replicate point that was nearest to the centroid of a cloud of all replicate points. The replicate point plotted in space that had the shortest distance to the centroid was chosen as the representative species to be used for further analyses. PCA plots of the replicates were generated for each species in R using ggbiplot with packages devtools and ggbiplot.

*Pteroplatytrygon* and *Rhina* with only 2 replicates could not be used for this method, therefore for these individuals I chose the specimen that was larger in overall body size (disc width) to be the representative.

### Size correction

To correct for differences in body size, a linear model was created where each measure was log scaled and regressed against a metric for body size. The statistical analyses were then performed on the size corrected residuals of the regression. Disc width or length is commonly used in interspecific studies to correct for body size in batoids. However, because of the great variance in disc shapes across species (ranging from round to triangular) and because of the comparatively very small measurements relating to the nose morphology, this did not seem appropriate. Head length would also not be appropriate due to the drastic differences in head shapes across batoids (i.e., the long snout of a small guitarfish has a larger head length than a much larger manta ray). Therefore, a new body size metric was created to minimize the effects of the broad differences in body shape across this group. This metric was measured from the last gill slit to the most anterior portion of the nostril, termed “gill to nare” length (GL), which accounts for differences in body size while not conflating measurements due to differing head shapes (Fig. 1).

### Phylogenetic ANOVAs and mapping

To determine if morphological differences were more than just the result of a shared lineage, I accounted for covariance due to shared ancestry by performing a phylogenetic principal component analysis and phyloANOVA. The phylogeny used was obtained from the open-source database “Vertlife.org” under the “shark subsets” where I was able to manually add each species. The database generated 100 phylogenies using a fully resolved 10-fossil set of 10K phylogenies from Stein et al. (2018). While the relationships were very similar across the 100 phylogenies, I chose the phylogeny that most closely resembled Aschliman et al. (2012), one of the most extensive batoid phylogenies to date. This phylogeny was then used to create a phylogram for ancestral state reconstruction and a phylomorphospace to visualize how nose and snout diversity evolved across Batoidea.

### Life history

To determine if ecology and other life history traits correlated with nose shape, size, and structure, I compiled data of possible relevant life history literature across the genera sampled here. Traits that may be relevant to olfaction and thus nose morphology included: lifestyle, habitat, swimming mode, and diet. Other metrics that I thought may be relevant to olfaction, including whether the species was migratory, would bury in the substrate, or segregate by sex, were not well-documented enough and ultimately too subjective to include with reasonable sample sizes. Therefore, the ecological metrics chosen here are well-established in the literature.

Lifestyle was categorized as benthic, demersal, bathydemersal, benthopelagic, and pelagic, following the categories and designations outlined in Last et al. (2016a). It should be noted that all categories have overlap of different degrees. Lifestyle categories represent a sliding scale of water depth where species are categorized by where they spend the majority of their time.

Habitat data were obtained via Fishbase (Froese and Pauly 2022) and Allen and Robertson (1994), Arkhipkin et al. (2008), Compagno (1999), Eschmeyer et al. (1983), Last et al., (2016a), and Micheal (1993). Habitat data were classified into 4 categories: soft/sandy bottom, rocky/reef bottom, open ocean, and deep sea. For habitat, many rays have some portion of their habitat with a soft/sandy substrate. Species here were categorized into the soft/sandy bottom category if they are exclusively found in this substrate type. Species were categorized into the rocky/reef bottom category if they are also found on rocky/reef substrate as noted by Fishbase. Only three species of batoids in this analysis were considered truly open ocean: the manta ray (*Mobula hypostoma*), the pelagic stingray (*Pteroplatytrygon violacea*), and the spotted eagle ray (*Aetobatus narinari*).

Swimming mode definitions followed traditional classification (Breder 1926; Webb 1998) with some recent modification including: body caudal fin (BCF), oscillatory, undulatory, intermediate, and a benthic locomotion “punting.” Designations into swimming mode categories follow Breder (1926), Koester and Spirito (2003), Macesic and Kajiura (2010), Rosenberger (2001), Rosenberger and Westneat (1999), Schaefer and Summers (2005), Webb (1998), and Wilga and Lauder (2002). Swimming mode also has varying degrees of overlap, as most batoid locomotion presents on a continuum between undulation and oscillation (Rosenberger 2001). Batoids were classified as undulatory (>1 wave) or oscillatory (<1/2 wave) based on the number of waves present along their pectoral fin. Batoids between these categories were classified as intermediate. Batoids that distinctly use axial-based locomotion via their caudal fin/tail were classified as body-caudal-fin (BCF) swimmers. Batoids that are also capable of true “punting,” a type of benthic locomotion associated with the pelvic fins, were classified as punters (Koester and Spirito 2003; Macesic and Kajiura 2010). While punting is not a true swimming mode, it is a method of slow propulsion that could be relevant to odor capture. “True punting” was defined in Macesic and Kajiura 2010 as batoids that engage only their pelvic fins during benthic locomotion (and not their disc or caudal fin). In this study, batoids were only classified as punters if they displayed this true punting locomotion, and not “augmented punting” seen in many benthic batoid species.

Diet follows the extensive batoid diet database outlined in Rutledge et al. (2019) that designates diet by biomechanical processing differences, included here were: soft prey (worms, fish), molluscivory (hard bivalves), and crustivory (shelled crustaceans). If diet data were not available for the exact species in question, the diet was inferred based on the diet of its closest intrageneric relative.

### Statistical analyses

To test if nasal morphology varied with differing life histories in batoids, a one factor analysis of variance (ANOVA; α = 0.05) was performed for each life history metric. An ANOVA was performed on each of the 18 measurement variables (PDM, PDX, etc.) for each life history metric (lifestyle: 5 levels, habitat: 4 levels, swimming mode: 5 levels, and diet: 3 levels.) For significant measurements, a post hoc Tukey honest significant differences test was run. ANOVAs were run separately on the size corrected data and again on phylogenetically corrected data (PhyloANOVAs) to understand how shared ancestry influenced the results.

Principle component analyses (PCA) and linear discrimination analyses (LDA) were also completed to visualize nasal morphotypes and correlations with life history. These analyses were performed on all 16 phylogenetically and size corrected continuous measurement traits listed in the morphometric model (therefore excluding two measurements that were count data: (PAN) proximal angle of nare and (HNA) head narial angle).

While useful, dimensionality reductions can exclude important traits for delimitation by focusing only on the axes that explain the most variation (Uyeda et al. 2015; Cadena et al. 2018). Therefore, a normal mixture model (NMM) analysis, that uses automatic variable selection with no *a prior*i information about delimitating groups, was performed. NMMs assume no information about group designation and instead identifies the number of groups (i.e., morphotypes) and assigns individuals to groups based on the number of distinct normal distributions in the phenotypic data.

All analyses were performed in R studio (R Core Team 2019) with packages: ggplot2, ape, phytools, geiger, plyr, ggpubr, ggbiplot, devtools, factoextra, MASS, picante, geomorph, tidyverse, magrittr, ggally, clustvarsel, raster, rasterVIS, RColorBrewer.

## Results

### Life History ANOVAs

Eleven of the 18 morphometric measurements were found to be significantly different between the life history metrics tested (swimming mode, diet, habitat, and lifestyle). These include: HWN, NDMX, ANFM, NSTRL, INM, ANF, AAW, ANW, PAN, HNA, NDMM (see methods for terminology). Of these metrics, 5 of the 11 remained significant with life history: HWN, NDMX, AAW, INM, and PAN (Table 2). These traits include the position and angle of the nostril on the head, the width of the inlet hole, and the spacing of the nostrils from each other. All 5 of these measurements were found to be significant between swimming mode, while 1 (NDMX) was also found to be significant in the lifestyle category. After phylogenetic correction, there were no significant differences in these measurement traits in the habitat or diet categories.

**Table 2:** Significant nasal measurements with their corresponding life history variables. Bolded measurements remained significant with phylogenetic correction. Acronyms are as follows: head width at narial opening (HWN), distance from nostril to disc margin maximum (NDMX), anterior nasal flap to mouth distance (ANFM), anterior aperture width/nare opening (AAW), exposed nostril length (NSTRL), distance across anterior nasal apertures (INM), anterior nasal flap base length (ANF), anterior nasal flap distal width (ANW), proximal angle of nare (PAN), head nostril angle (HNA), distance from nostril to disc margin minimum (NDMM).

Life History Variable	Significant Measurement Trait
swimming, diet	**HWN**
swimming, lifestlye	**NDMX**
swimming	ANFM
swimming	**AAW**
swimming	NSTRL
swimming	**INM**
swimming	ANF
swimming	ANW
swimming	**PAN**
swimming	HNA
diet	NDMM

#### 1A. Swimming mode

With 5 swimming mode levels and 10 significant morphometric measurements, there were 25 significant pairs (See Table 3 for pairs and *P*-values). The nasal measurements associated with BCF swimmers were significantly different from all other swimming modes (oscillatory, undulatory, and true punters). Specifically, metrics relating to the size and shape of the inlet hole (AAW, NSTRL), the anterior nasal flap (ANFM), the placement (angle and distance from disc edge) of the nostril on the head (PAN, HNA, INM, NDMX) were all significant metrics that distinguished BCF swimmers from the other swimming modes. However, after correcting for shared ancestry, half of these variables remained significant: HWN, NDMX, AAW, INM, and PAN.

**Table 3:** Significant pairs of ecological traits for ANOVAs and PhyloANOVAs. Ecological traits are classified by swimming mode, lifestyle, and diet. There were no significant values for habitat. If measurement values remained significant after phylogenetic correction, they are bolded here. Residual mean pair 1 refers to the size corrected mean of the first listed ecological trait for that measurement in the pairwise comparison. Residual mean pair 2 refers to the second trait in the listed pair. The metrics PAN and HNA were angle measurements and therefore not size corrected.

Ecological Trait	Significant Pair	Measurement	*P*-value	phylo *P*-value	Pair 1	Pair 2
Swimming Mode	**oscillatory-intermediate**	**HWN**	**0.048**	**0.020**	–0.318	0.267
	oscillatory-BCF	NDMX	0.031	0.680	–0.699	0.239
	**oscillatory-intermediate**	**NDMX**	**0.020**	**0.030**	–0.699	0.384
	intermediate-BCF	ANFM	0.006	0.230	–0.916	1.461
	oscillatory-BCF	ANFM	0.004	0.256	–0.906	1.461
	undulatory-BCF	ANFM	0.021	0.234	–0.577	1.461
	intermediate-BCF	AAW	0.016	0.315	–0.061	0.625
	oscillatory-BCF	AAW	0.001	0.136	–0.220	0.625
	**punt-BCF**	**AAW**	**0.004**	**0.030**	–0.169	0.625
	**undulatory-BCF**	**AAW**	**3.25E-04**	**0.030**	–0.381	0.625
	punt-BCF	NSTRL	0.049	0.320	–0.705	0.661
	**undulatory-BCF**	**INM**	**0.008**	**0.040**	–0.345	0.369
	intermediate-BCF	ANF	0.028	0.630	0.290	–0.480
	oscillatory-BCF	ANF	0.017	0.630	0.299	–0.480
	intermediate-BCF	ANW	0.002	0.120	0.618	–1.176
	oscillatory-BCF	ANW	0.001	0.126	0.610	–1.176
	punt-BCF	ANW	0.051	0.217	0.062	–1.176
	undulatory-BCF	ANW	0.020	0.160	0.234	–1.176
	**intermediate-BCF**	**PAN**	**1.87E-04**	**0.016**	105.4	37.3
	**oscillatory-BCF**	**PAN**	**1.87E-04**	**0.035**	102.0	37.3
	**punt-BCF**	**PAN**	**2.63E-04**	**0.010**	103.6	37.3
	**undulatory-BCF**	**PAN**	**3.22E-05**	**0.010**	114.8	37.3
	oscillatory-BCF	HNA	0.020	0.544	114.0	126.6
	punt-BCF	HNA	0.041	0.306	114.6	126.6
	undulatory-BCF	HNA	0.021	0.210	113.4	126.6
Lifestyle	**demersal-benthopelagic**	**NDMX**	**0.010**	**0.030**	0.520	–0.740
	**demersal-pelagic**	**NDMX**	**0.018**	**0.030**	0.520	–0.650
Diet	crustacivory-molluscivory	HWN	0.043	0.084	0.120	–0.360
	soft prey-molluscivory	HWN	0.044	0.084	0.060	–0.360
	soft prey-molluscivory	NDMM	0.033	0.063	0.150	–0.670

Oscillatory, undulatory, and punting swimmers were found to have nostrils positioned closer to the edge of their head (NDMX) compared to BCF and intermediate swimmers. However, only oscillatory and intermediate swimming modes were significantly different from each other before and after phylogenetic correction. Specifically, oscillatory swimmers had a shorter distance from their nostril to disc margin (NDMX) compared to all other swimmers but were significantly different between intermediate swimmers only (*t = 3.37, P = 0.03*). Oscillatory swimmers also had shorter head widths at their nostrils (HWN) compared to intermediate swimmers (*t = 2.97, P = 0.02*).

BCF swimmers were found to have the greatest distance from their anterior nasal flap (the nasal curtain that covers the excurrent outlet in most rays) to their mouth (ANFM) than undulatory, oscillatory, and intermediate swimmers. The width of the anterior nasal flap (ANF) was also much smaller in BCF swimmers, with oscillatory and intermediate swimmers having the widest anterior nasal flap. Oscillatory and intermediate swimmers were found to have the shortest distance from this flap to their mouth (ANFM). The exposed nostril length (NSTRL) was also much larger in BCF swimmers compared to all others, with oscillatory and intermediate swimmers having the smallest nostril length. However, these traits did not remain significantly different with phylogenetic correction.

BCF swimmers were found to have their nostrils spaced farther apart (INM) than other swimmers, while undulatory swimmers had their nostrils positioned the closest together. This was significantly different in BCF vs. undulatory swimmers (*t = 3.75, P = 0.04*) after phylogenetic correction. Anterior aperture width, the diameter of the inlet hole (AAW), was found to be significantly different after phylogenetic correction in punters vs. BCF swimmers (*t = 4.50, P = 0.03*) and undulatory vs. BCF swimmers (*t = 5.09, P = 0.03*) with both punters and undulatory swimmers having smaller inlet hole diameters than BCF swimmers. Interestingly, oscillatory swimmers were not found to be significantly different from BCF swimmers in this metric.

The angle of the nostril with respect to the head/gill position (HNA) was greater in BCF swimmers compared to all other swimmers (126° vs. 114°), however, this metric was not significant after phylogenetic correction. But, the proximal angle of the nare (PAN) was found to be significantly different from all swimming modes after phylogenetic correction when compared to BCF swimmers: undulatory vs. BCF (*t = −6.06, P = 0.01*), punters vs. BCF (*t = −5.32, P = 0.01*), intermediate vs. BCF (*t = −5.32, P = 0.016*), and oscillatory vs. BCF (*t = −5.32, P = 0.035*). Intermediate, oscillatory, and punters had nostrils positioned between ∼102°–105°, while undulatory swimmers had nostrils positioned at ∼114° and BCF swimmers’ nostrils were positioned at ∼37°.

#### 3A. Lifestyle

With five lifestyle levels and one significant morphometric measurement, there were two significant pairs (Table 3; Supplementary Table 1). The position of the nostril on the head (NDMX) was significantly different when comparing demersal batoids to both benthopelagic and pelagic batoids. This remained significant after phylogenetic correction with benthopelagic (*t = −3.69, P = 0.03*) and pelagic batoids (*t = −2.29, P = 0.03*) with nostrils positioned closer to the edge of their disc and head compared to demersal batoids.

#### 4A. Habitat and diet

There was no significant difference in any nasal measurements between the four habitat levels tested (soft/sandy, rocky/reef, open ocean, and deep sea). Rocky/reef animals and open ocean animals were close to significance for the NDMX metric (*P = 0.08*); however, this was not near significance after phylogenetic correction.

With three diet levels and two significant morphometric measurements, there were three significant pairs (Table 3). These traits related to head size and nostril position on the head (HWN, NDMM). Molluscivore batoids like eagle rays and bowmouth guitarfish that eat hard-shelled prey were found to have narrower head widths at their nostrils compared to the other feeding modes. Molluscivores were also found to have nostrils that were positioned closer to the edge of their head than batoids that consume soft prey. However, these traits did not remain significant with phylogenetic correction. The metric NDMM was close to significance between soft prey eaters and molluscivores after phylogenetic correction (Table 3; *t = −2.68, P = 0.06*).

### Phylogenetic analysis

**Fig. 2:**
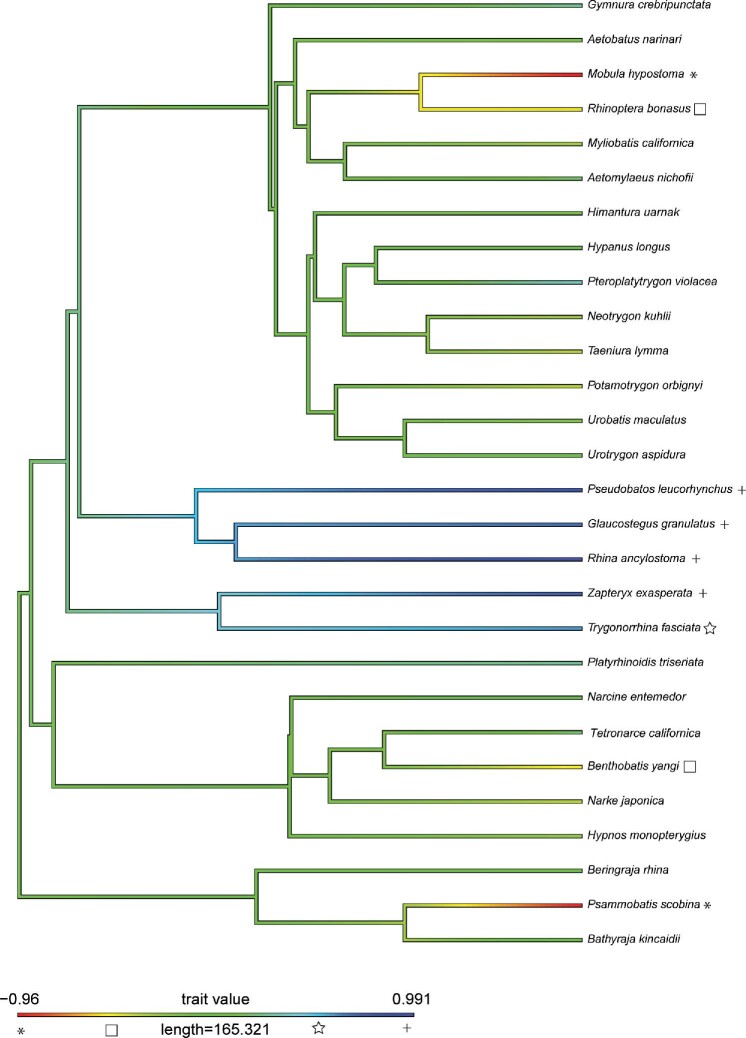
Phylogram showing the evolutionary relationships (based on Aschliman et al. 2012) of all 28 species in this study. The trait value heat map shows intermediate trait values in green (that are reconstructed to be ancestral) and evolutionary divergence in red or blue. Here, most guitarfishes are highlighted in dark blue (+) except for one , a skate and manta ray in red (*), and cownose ray and blind electric ray in yellow, suggesting divergent nasal features in these groups. Symbols added for colorblind assistance.

**Fig. 3:**
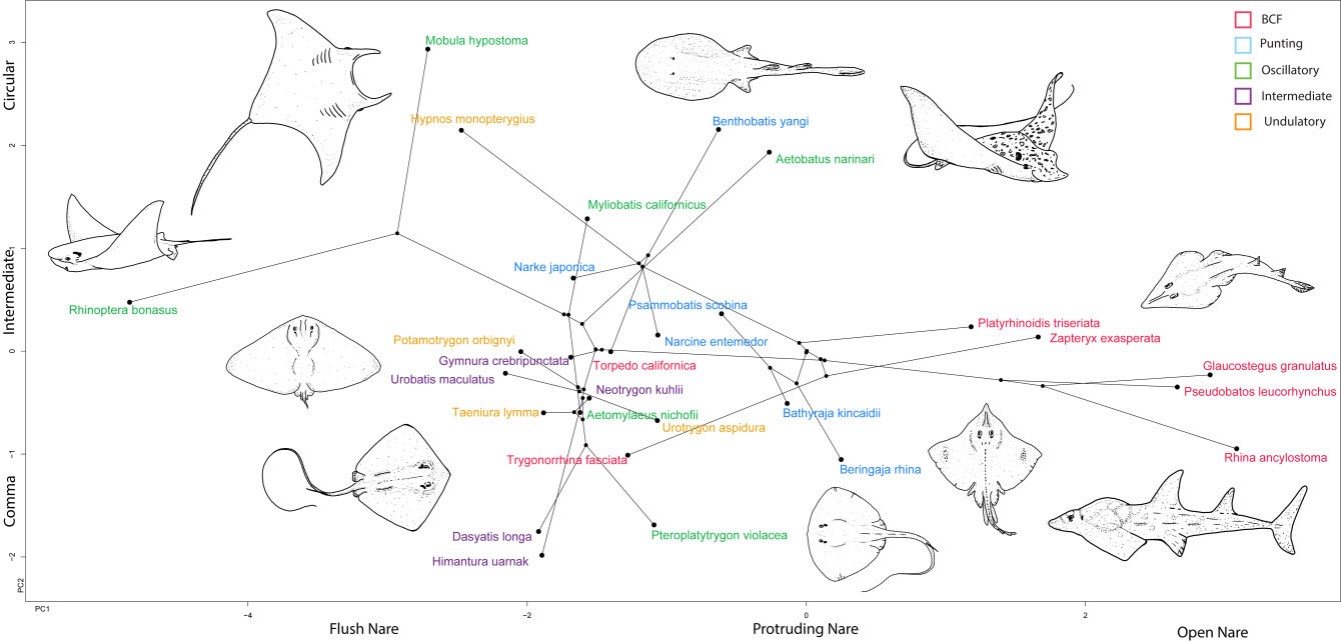
Phylomorphospace (relationships based on Aschliman et al. 2012) showing the results of the PhyloPCA morphometric measurements on nasal morphology colored coded by swimming type. Morphotypes overlaid on axes and representative batoids illustrated in the morphospace. Long branches extending across the phylomorphospace indicate convergent evolution.

The phylogram and phylomorphospace highlighted some evolutionary trends. In the phylogram, the ancestral values can be seen in green, while areas of divergence are in blue, red, and yellow. Most of the Rhinopristiformes (excluding *Trygonorhinna*), or guitarfishes, are clustered in blue, while *Psammobatis* (sand skate), *Mobula* (manta ray) are in red, and *Benthobatis* (blind ray) and *Rhinoptera* (cownose ray) are in yellow, suggesting possible evolutionary convergence or parallelism of these corresponding nasal features (Fig. 2). The phylomorphospace also showed distinct grouping in space, with long branches across the phylogeny, specifically with the open nare morphotype on the far right and the flush nare on the far left (Fig. 3).

### Dimensionality reductions

**Fig. 4:**
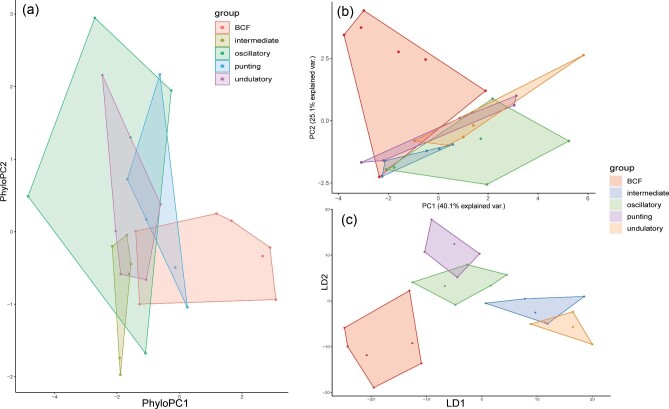
Results of nasal morphology grouped by swimming mode for (a) phylogenetically corrected principal component analysis, (b) standard principal component analysis, and (c) linear discriminant analysis. Axis loadings are listed in Table 4.

**Fig. 5:**
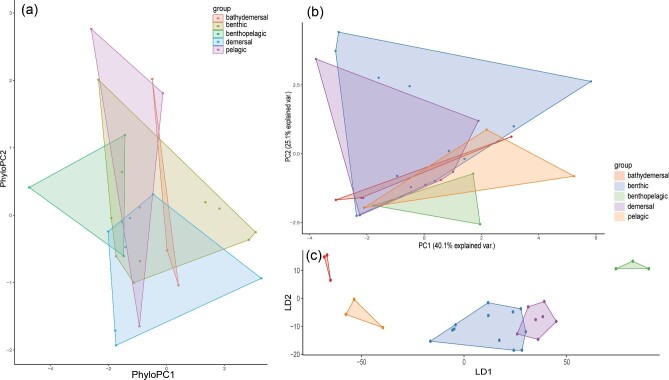
Results of nasal morphology grouped by lifestyle for (a) phylogenetically corrected principal component analysis, (b) standard principal component analysis, and (c) linear discriminant analysis. Axis loadings are listed in supplementary.

**Fig. 6:**
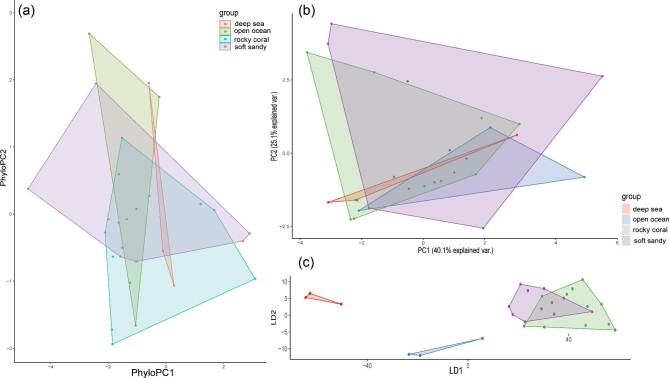
Results of nasal morphology grouped by habitat for (a) phylogenetically corrected principal component analysis, (b) standard principal component analysis, and (c) linear discriminant analysis. Axis loadings are listed in supplementary.

**Fig. 7:**
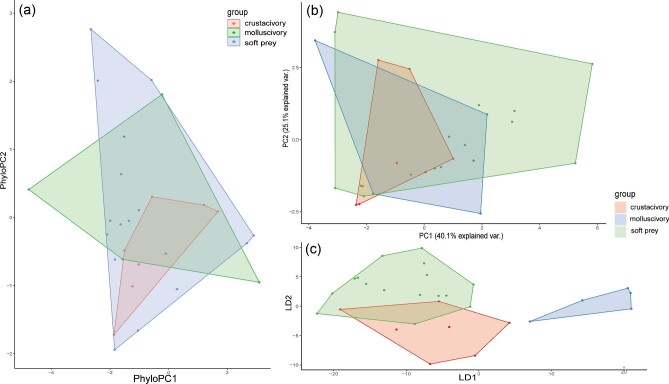
Results of nasal morphology grouped by diet for (a) phylogenetically corrected principal component analysis, (b) standard principal component analysis, and (c) linear discriminant analysis. Axis loadings are listed in supplementary.

Swimming mode was found to be the most relevant ecological metric to correlate with nose morphology and an in-depth description of the swimming mode dimensionality reductions are described below. The other ecological metrics (habitat, lifestyle, and diet) were not as informative once phylogenetically corrected (Fig. 4–7). However, there were some interesting observations. Benthic and demersal batoids generally occupied larger regions of the morphospace, suggesting more nose diversity in very ground-associated ecologies (Fig. 5). While deep sea and bathydemersal species occupied the narrowest region of morphospace. The LDA for lifestyle found that benthic species occupy the largest region of morphospace (Fig. 5). Demersal species occupied a smaller but overlapping region in the benthic morphospace. Pelagic and bathydemersal batoids occupied another distinct but partially overlapping region of the morphospace, with benthopelagic species occupying the opposite end of the morphospace (Fig. 6). All four habitat types were in complete overlap, with soft sandy habitat and rocky reef occupying the largest morphospace, and open ocean and deep sea occupying smaller regions (Fig. 6). The LDA for habitat was not able to discriminate between soft sandy and rocky reef habitat types, but open ocean and deep-sea habitats were in distinct regions in the morphospace, suggesting these nose morphologies are less diverse or possibly more specialized. Batoids that eat soft prey occupied the largest region of morphospace and overlapped with crustacivores (Fig. 7). While the LDA for diet revealed that molluscivores can be mostly discriminated in the morphospace, but still had partial overlap with crustacivores (Fig. 7). All PCA, PhyloPCA, and LDA loadings for habitat, lifestyle, and diet ecologies can be found in the supplementary.

#### 1C. Swimming Mode Principle Component Analysis

The first two PCs accounted for 65.2% of the total variance (Fig. 4). PC1 primarily loaded with traits relating to the position of the nostril in relation to the mouth (NOM, NOX), the distance from the nostril to the most anterior region of the snout (PDM, PDX), the spacing of the nostrils (INM), the size of the inlet hole (AAW) and total exposed nostril length (NSTRL). These traits all loaded negatively. PC2 primarily loaded with traits relating to the anterior nasal flap, also known as the nasal curtain (ANF, ANW, ANL), as well as the distance from this flap to the mouth (ANFM), and the spacing between the outlet nasal chambers (INW). ANF, ANW, and ANL all loaded negatively while ANFM and INW loaded positively. See Table 4 for a complete list of PC1-4 loadings, accounting for 87.9% of the total variance. In the morphospace, we can see BCF swimmers loaded in the top left, while oscillatory swimmers loaded in the bottom left, with almost no overlap. Undulatory, intermediate, and punting batoids all loaded in the middle. Intermediate swimmers did not overlap with undulatory or punting batoids but were in the oscillatory space with some overlap in BCF swimmers. Undulatory and punters had a fair amount of overlap, but both extended farther into opposite regions of the morphospace.

**Table 4:** Loadings of the first 4 PCs for the PhyloPCA and standard PCA (accouting for 83% and 88% of total variance, respectively,) and LDA (100% total variance) based on swimming mode and benthic locomotion for nasal morphology.

	PhyloPC1 (33.3%)	PhyloPC2 (28.2%)	PhyloPC3 (12.8%)	PhyloPC4 (8.5%)		PC1 (40.1%)	PC2 (25.1%)	PC3 (16.2%)	PC4 (6.5%)		LD1 (49.4%)	LD2 (30.7%)	LD3 (13.3%)	LD4 (6.6%)
**PDM**	0.3584154	−0.73106	−0.32922	−0.17423	**PDM**	−0.3662	0.04277	−0.015	−0.0929	**PDX**	−5.8775	5.38019	−0.097	−5.641
**PDX**	0.2969774	−0.79571	−0.2877	−0.24635	**PDX**	−0.3661	−0.0138	0.00842	−0.1765	**NDMM**	−5.8267	4.20162	−0.1314	−2.6832
**TDW**	−0.354549	−0.01288	0.191578	0.621617	**TDW**	0.1497	−0.0353	0.25069	0.73888	**NOX**	−4.2282	13.9905	5.34926	−5.0602
**HWN**	−0.069644	−0.81471	0.113865	0.386129	**HWN**	−0.2169	−0.1271	0.454	0.0979	**NOM**	−2.781	−6.8678	−3.4465	5.55828
**NOM**	0.091308	−0.7559	−0.51496	−0.07723	**NOM**	−0.305	−0.2583	−0.0741	−0.0618	**INM**	−2.6928	−12.98	−3.8538	5.81279
**NOX**	0.332519	−0.69745	−0.30918	−0.32571	**NOX**	−0.3668	0.02301	−0.1609	0.05602	**AAW**	−−2.4437	0.67281	−1.494	−3.2768
**NDMM**	−0.265482	−0.81828	0.250002	0.281995	**NDMM**	−0.0987	−0.1793	0.54049	−0.1477	**ANF**	−1.5483	4.17596	1.03328	−1.164
**NDMX**	−0.017161	−0.89367	0.330226	0.185599	**NDMX**	−0.2245	−0.0016	0.49494	−0.1011	**TDW**	−1.1788	0.50317	−0.4022	1.20264
**ANFM**	0.9452175	0.099712	−0.15401	0.237191	**ANFM**	−0.2017	0.32414	−0.0925	−0.0207	**ANFM**	−0.4606	0.68761	0.52935	0.47914
**AAW**	0.4477639	−0.62472	0.001496	0.064315	**AAW**	−0.2937	0.2262	0.04218	0.29185	**NSTRL**	1.0319	−1.7538	−0.9231	−0.4729
**NSTRL**	0.3707298	−0.59127	0.340937	−0.26053	**NSTRL**	−0.2934	0.12316	0.03765	0.01334	**ANW**	1.65861	−2.6363	−0.6476	1.29334
**INM**	0.3992996	−0.3775	−0.41266	0.054243	**INM**	−0.3077	0.06441	−0.2209	0.38583	**INW**	1.70149	7.7058	2.10868	−10.95
**INW**	−0.085349	−0.31012	−0.82969	0.189393	**INW**	−0.1808	−0.3627	−0.2163	0.28184	**ANL**	2.54744	−1.5242	−1.2392	2.45297
**ANF**	−0.366511	−0.25949	−0.8159	0.226796	**ANF**	−0.0331	−0.4695	−0.0918	0.09302	**HWN**	3.26275	2.67096	2.30476	2.9632
**ANL**	−0.18226	−0.44709	−0.54744	−0.4134	**ANL**	−0.1494	−0.3699	−0.2113	−0.2033	**NDMX**	5.87935	−5.8719	1.35638	0.34538
**ANW**	−0.703215	−0.1649	−0.51874	0.226268	**ANW**	0.10449	−0.4657	0.0431	0.04066	**PDM**	7.11214	−6.7245	−0.3669	7.25542

#### 2C. Swimming Mode Phylogenetic Principle Component Analysis

The phylogenetic PCA resulted in grouping patterns quite similar to the standard PCA, but with more overlap (Fig. 4, Table 4). PC1 and PC2 loaded with the same traits as described in the standard PCA. The first two PCs accounted for 61.5% of the total variance. The first four PCs accounted for 82.8% of the total variance. The major difference between the two PCAs is that now the undulatory swimmers’ noses fully overlap with the oscillatory swimmers’ noses. Punters are now distributed evenly between BCF and oscillatory swimmers in the morphospace, while the noses of intermediate swimmers lie mostly in the oscillatory space.

#### 3C. Swimming Mode Linear Discriminant Analysis

The linear discriminant analysis found that the traits that best distinguish nasal morphology by swimming were relating to the distance from the nostril to the most anterior region of the snout (PDX, PDM), the spacing of the incurrent and excurrent nostrils from each other (INM, INW), the distance from the incurrent nostril to the mouth (NOX), and the distance from the nostril to the edge of the head/disc (NDMX). The first two LDs accounted for 80% of the total variance. See Table 4 for a complete list of LDA 1–4 loadings.

### Normal Mixture Models

**Fig. 8:**
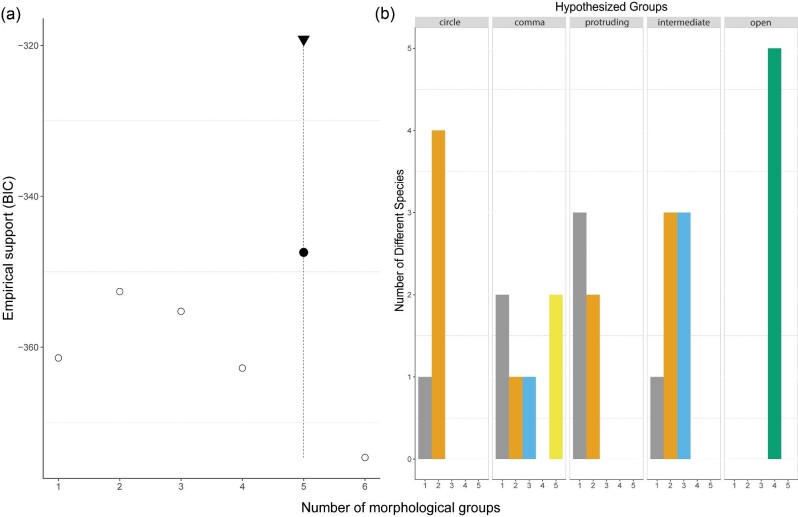
Results from the Normal Mixture Model analysis that assumes no a priori information about groups. (A) graph showing the BIC score and corresponding model (VII) with the highest empirical support identified 5 distinct morphotypes in these data. (B) histogram comparing the assignment of individuals to groups between the best Mclust models and the hypothesized morphotypes (open, flush [circle, comma, intermediate], and protruding).

The first three principal components were the most useful for group discrimination (number of morphotypes) in the Normal Mixture Models (NMMs). The mixture model that specified five morphological groups received the strongest support (Fig. 8a). The gaussian finite mixture model identified a Bayesian Information Criterion (BIC) for each of the 16 possible models. The model with the highest BIC score was spherical, unequal volume (VII) with a BIC score of −347.4 (*log-likelihood: −2.03, n = 28, ICL = −377.26*). The clustering table found that of the five identified morphological groups, there were 10, 7, 5, 4, and 2 individuals in each group (Fig. 8b). I hypothesized that there would be 7, 6, 5, 5, and 5 individuals in each group (see the following section on morphotype classification and limitations in discussion section). The second-best model was the diagonal, varying volume, equal shape (VEI) with BIC score of -352.6. The model identified only two morphological groups. However, the best model (VII) had a difference of 6 points in BIC score between the second-best model (VEI). BIC scores with differences of six or greater are regarded as strong evidence against lower support models (Kass and Raftery 1995). Therefore, there is strong support for the VII model which parsed out five groups or distinct morphotypes from these data.

### Morphotype classification

Based on visual examination, statistical significance of measurement traits, and the results of the normal mixture models, there appears to be five morphological groups, or morphotypes, found in batoid nasal diversity. Here I suggest a morphological classification system that can be used when describing batoid nasal morphology with ecological and likely hydrodynamic implications (see discussion section). Batoid nasal diversity can be classified into three major morphotypes that can be further subdivided into five total morphological groups.

**Fig. 9:**
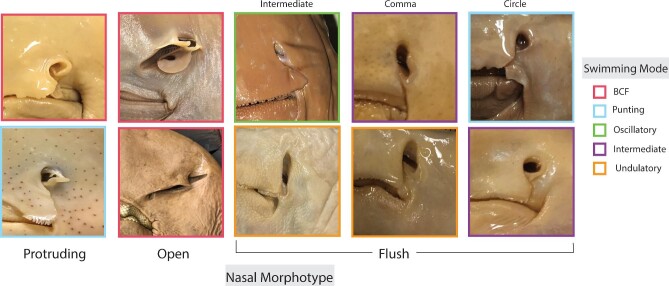
The major nasal morphotypes identified in this study: protruding, open, and flush. Flush has 3 subcategories including intermediate, comma shaped, and circular. From top left going down each row: Tetronarce california, Beringraja rhina, Pseudobatos leucorhynchus, Rhina ancylostoma, Rhinoptera bonasus, Potamotrygon orbignyi, Urobatis maculatus, Urotrygon aspidura, Narcine entemedor, Gymnura micura. Color coded based on swimming mode.

The first and most common nasal morphotype is termed the “flush” morphotype, where the incurrent nostril (AAW) is flush on the head of the animal, with no external protrusions. This morphotype can be further subdivided into categories based on the shape of the inlet hole: circle, comma, or intermediate (slit) shaped. This is the most diverse morphotype and can be accompanied with a nasal curtain of square, skirt, or incomplete type (see methods for descriptions). The circle shape can be seen in some myliobatids, dasyatids, narcinids, and gymnurids (Table 1). To be classified as a circle morphotype, the incurrent nostril width should be approximately as wide as it is long. The representative species for the flush circle morphotype is *Narcine entemedor* with a square nasal curtain (Fig. 9).

The next subdivision of the flush morphotype, is the comma shape, which describes an inlet opening that resembles a comma or curved kidney bean. The comma shape can be seen in many different species of dasyatids and urotrygonids. To be classified as a comma morphotype, the incurrent nostril opening should be longer than it is wide and narrowest in the medioposterior region of the inlet with a distinct, identifiable constriction. The representative species for the comma shape is *Urobatis maculatus* with a skirt shaped nasal curtain.

The last subdivision of the flush morphotype is the intermediate shape, which describes an oval inlet shape or narrow, slit like opening. The intermediate shape can be seen in some rhinopterids, dasyatids, and potamotrygonids. To be classified as an intermediate morphotype, the incurrent nostril should be longer than it is wide, with approximately the same width along the length of the inlet, without the distinct constriction seen in the comma morphotype. This can look like the angled oval inlet shape seen in *Rhinoptera* and *Aetobatus*, or long and skinny like in *Potamotrygon*. The representative species for the intermediate shape is *Rhinoptera bonasus* with a skirt shaped nasal curtain (Fig. 9).

The next morphotype is the easily identifiable “open” nare morphotype, that was named due to its absence of a closed nasal chamber and excurrent channel. In the open morphotype, the incurrent and excurrent regions of the nostril are not distinct, and only partially divided by the anterior nasal flap (ANF). The nasal lamellae, which are thin sheets of sensory tissue that line the internal nasal chamber in batoids, are easily visible and fully exposed to the environment. The open nare morphotype is exclusively seen in Rhinopristiformes, the guitarfishes and sawfishes. However, not all guitarfishes have this morphotype, as *Trygonorhinna* has a nasal curtain that separates the inlet from the outlet. This morphotype is accompanied with either a reduced or square nasal curtain. The representative species for the open nare morphotype is *Rhina ancylostoma* with a reduced nasal curtain (Fig. 9).

The last morphotype is termed the “protruding morphotype” that describes a protruding structure that partially or fully encloses the incurrent nostril. This morphotype can be easily identified by looking at the batoid from the side, laterally, to determine if the nostril protrudes downwards from the body. The protruding morphotype can be seen in torpedids, narkids, and Rajiformes. The protruding morphotype can be either cylindrical, conical, or cupped. Cylindrical nostrils are seen in some torpedo rays, where the inlet hole is fully enclosed in a fleshy, protruding tube. Conical or cupped nostrils are seen in skates, where the inlet hole is flanked with a protruding flap that can be cone or paddle shaped that extends only partially around the circumference of the inlet opening. This morphotype is accompanied with either an incomplete or square nasal curtain. The representative species for the protruding morphotype is *Beringraja rhina* with an incomplete nasal curtain (Fig. 9).

## Discussion

This study is the first to quantitatively describe and broadly classify batoid nasal diversity within an ecological and phylogenetic framework. While batoids have impressive olfactory abilities (Meredith et al. 2012) and an expansive nasal morphology, there has been little research into the morphology and biomechanics of their olfactory system, specifically the external odor-harnessing morphology. The morphometric measurements that best distinguish batoid nasal morphology were traits related to the position and angle of the nostrils on the head (NDMX, NDMM, PAN, HNA), the diameter of the inlet and total exposed nostril (AAW, NSTRL), the spacing of the nostrils from each other (INM), the size and shape of the nasal curtain (ANF, ANW), and the distance from the nasal curtain to the mouth (ANFM). This study specifically identified five major morphotypes seen in Batoidea and found that swimming mode was the ecological metric that correlated best with batoid external nasal diversity, in terms of statistical significance and distinct groupings in the phylomorphospace.

Swimming mode (BCF, undulatory, oscillatory, intermediate, and punting) correlated best with nasal morphology, resulting in five significant morphometric measurements with phylogenetic correction: HWN, NDMX, AAW, INM, and PAN. Oscillatory swimmers (like the eagle rays, bat rays, manta rays) and BCF swimmers (guitarfishes, sawfishes) had narrower heads with nostrils positioned closer to the edge of their disc. BCF swimmers generally had larger incurrent nostril diameters (AAW) when compared to both undulatory and punting batoids, like many Dasyatid stingrays. Oscillatory swimmers were also distinct from intermediate swimmers, with their nostrils positioned closer to the edge of their disc. BCF swimmers also had their nostrils positioned farther apart on their head and positioned at a smaller angle on the head when compared to all other swimmers. These results were slightly unexpected, as generally BCF and oscillatory swimmers had statistically similar traits but were still in distinct regions of the morphospace. This is likely because BCF swimmers could be discriminated from oscillatory swimmers due to differences in the nasal curtain (or anterior nasal flap), but in terms of the inlet hole shape, size, and position, were similar to each other. This could be because compared to the other swimmers, oscillatory and BCF swimmers may operate at higher Reynolds numbers (Re = *uL/ν*, where *u* is the fluid velocity, L is the characteristic linear dimension, and *ν* is the kinematic viscosity). Many oscillatory and BCF swimmers are some of the largest batoids, with larger inlet hole diameters (even with size correction) and swim at fasters speeds than many of their counterparts. However, swimming mode does not correlate directly with velocity, and if it did, oscillatory swimmers would likely be statistically significant from all other swimming modes, which it was not. The motion in which the animal swims, and thus directs odors into its nose, may also influence odor capture. For example, BCF swimmers move their heads in a wide lateral sweeping motion (i.e., yawing), which may help entrain and flush their more horizontally expanded nostrils. Oscillatory swimmers, that swim with a more vertical up and down motion (i.e., pitching), have more vertically oriented nostrils that are oval or comma shaped. Undulatory swimmers are generally more dynamic swimmers, capable of quick movements and turns, and displayed a diversity of flush nostril types. Overall, of the proposed morphotypes, BCF swimmers were found to possess the open nare morphotype, punters had the protruding nare morphotype, and oscillatory, undulatory, and intermediate swimmers had the flush morphotype. Within the flush morphotype, oscillatory and undulatory swimmers generally had comma or intermediate shaped inlets, and intermediate swimmers had circle or intermediate inlet shapes.

Habitat (soft/sandy, rocky/reef, deep sea, open ocean) was not a relevant metric for discriminating external nasal morphology in batoids. This was surprising because elasmobranchs that live in the open ocean and deep-sea have been shown to have the largest olfactory bulbs, a reliable proxy for olfactory sensitivity, indicating that a heightened sense of smell may be important for these ecologies (Yopak et al. 2015, 2019). Additionally, reef associated species were found to have the smallest olfactory bulbs but enlarged optic tecta (a region of the brain associated with visual cues). While there was no statistical difference in the measurement traits of habitat groups in this study, both deep sea and open ocean batoids did show discrimination in the linear discriminant analysis (LDA), both in distinct regions of the morphospace from the overlapping rocky reef and soft sandy habitats. Both deep sea and open ocean species represented much smaller regions of the morphospace, suggesting less diversity in external nasal features. However, there was also fewer representative species in both of these categories. The deep sea and open ocean species in this study generally had more circular inlet holes, with the circle or protruding nare morphotypes, and most commonly, the incomplete nasal curtain. Whether this circular inlet shape confers any sort of odor-harnessing advantage over other inlet shapes (comma, slit shaped) is unknown. Open ocean batoids, that generally swim at faster speeds, may not require a more specialized odor capturing morphology. Many deep-sea species, like skates, are generally slow moving and their protruding nare morphotype may help to capture odor in these slow flow environments.

The five lifestyle categories (benthic, demersal, bathydemersal, benthopelagic, and pelagic) following Last et al. (2016b) resulted in only one statically significant morphometric measurement: NDMX, the distance from the medial edge of the incurrent nostril to the disc. Specifically, demersal rays were found to have their nostrils positioned more medially on their head, farther away from the edge of their disc when compared to both benthopelagic and pelagic rays. Benthopelagic and pelagic batoids included the pelagic stingray, eagle rays, bat rays, cownose rays, and manta rays. Previous research looking at the lamellae count of benthopelagic elasmobranchs found that on average they have 93 lamellae, as opposed to an average of 56 in benthic species (Meng and Yin 1981). However, as previously noted, lamellae count alone is not a reliable proxy for olfactory sensitivity. The myliobatid rays in this open ocean group (manta ray, eagle ray, bat ray, and cownose ray) also have some of the largest brains of all elasmobranchs, with an enlarged telencephalon (Northcutt 1978), which, like the olfactory bulb, also processes olfactory information. The spotted eagle ray is also known to have a very large sensory surface area relative to other elasmobranchs, and an allometric relationship with lamellae count and internarial distance (INM), suggesting olfaction may be very important to this species throughout its life (Schluessel et al. 2010). These more open ocean, migratory batoids generally have their nostrils positioned closer to the edge of their disc and head. From a sensory perspective, it is likely more advantageous to have nostrils positioned more anterolaterally on the body, where it could be easier to receive a chemical cue. Contrary to the typical stingray body plan, many myliobatids also have more prominent, protruding heads and snouts, which is likely why the head widths at the nare (HWN) did not remain significant with phylogenetic correction. Regardless, a more sharply pointed head with nostrils positioned closer to the edge, may help quicken the time it takes for an odorant to reach the nostril (if already oriented into a plume). Overall, benthopelagic and pelagic rays were commonly observed with the intermediate or comma nare morphotype. These nostrils were generally oval in shape and the incurrent nostril was angled to a higher degree on the head. Demersal rays were generally more variable in morphology, with all five nare morphotypes observed in this group. The phyloPCA did show separation between demersal and benthopelagic rays, and the LDA was able to discriminate between benthopelagic, pelagic, and bathydemersal species.

Diet (molluscivory, crustacivory, soft prey) based on biomechanical processing differences, was found to be not very informative for discriminating nasal morphologies. It seemed possible that certain prey items (fish vs. bivalves) would require a greater degree of fine scale odor tracking that may influence the evolution of a more robust nasal morphology. Previous research looking at elasmobranch olfactory bulb mass found that crustacean eating elasmobranchs had lower bulb mass than mollusk and echinoderm eating elasmobranchs (Schluessel et al. 2010). However, the external morphology of batoids that eat different prey items did not reflect this. Without accounting for phylogeny, molluscivore batoids (eagle rays, bat rays, and bowmouth guitarfish) had nostrils positioned significantly closer to the edge of their head than batoids that consume soft prey. However, with phylogenetic correction, this pattern did not hold and there was much overlap in the morphospace between diet types. Interestingly, the LDA was able to distinguish molluscivores from the other two overlapping prey types, but this could be more related to the narrow head widths seen in myliobatids than other nasal features.

Based on the morphometric measurements of this study alone, the NMMs identified five major morphotypes seen in batoids. This model was able to identify the open nare morphotype easily. It was also able to identify a separate group inside the comma morphotype. However, all other groups had some discrepancy with hypothesized and realized groups. This is likely because these data did not include all relevant features including important angle measurements, as well as other defining traits such as nasal protrusion length and the complex shapes of the inlet hole. These data had to be omitted for this type of analysis. Specifically, measurements had to be applicable across all batoids and was therefore limited to features present in all batoids and known homologous features (like the anterior nasal flap and nasal curtain). Therefore, presence/absence traits like the additional nasal flaps of guitarfish and butterfly rays, and the conical projection of many skates and electric rays, could not be incorporated into this model. If these unique features could be better captured quantitatively, this may help explain additional variance between morphotypes. The measurements in this study aimed to be as discrete as possible to minimize subjectivity. The width and length of the incurrent nostril was measured, but this alone could not capture the variation in complex nostril inlet shapes. Angle data also could not be included in the NMMs analyses because it was count data, but it did show statistical significance across groups, suggesting this is an important character for discrimination. It may be beneficial for future studies to incorporate geometric morphometric analyses of nostril inlet shapes, however, picking repeatable points around the incurrent nostril may be challenging.

The proposed five major morphotypes all have functional implications for odor capture. Batoid olfaction is a hydrodynamic process, that relies upon odor to be directed into the nose. Without a direct pump inside the nasal cavity, the morphology of the system is likely crucial for successful odor capture. Additionally, an odorant molecule will have the challenge of overcoming the boundary layer surrounding a swimming fish: the thin layer of stationary fluid (*velocity = 0*) that encapsulates its body. This layer acts a barrier to odor transport. While the mechanisms for which odor is harnessed into the batoid nose has not been explored, it is hypothesized that batoid olfaction relies upon one or a combination of the following mechanisms: the motion pump, the buccopharyngeal pump, pressure differences, or some other form of unknown mechanical agitation (movement of jaw bones) (Settles 2005; Cox 2008). The motion pump is more likely to be observed in faster swimming animals with nostrils positioned directly in the path of water flow. The buccopharyngeal pump refers to the water that is drawn into the mouth of the animal during mouth-associated respiration (vs. spiracle only respiration). Because the outlet nostril channel of some batoids is positioned directly above the mouth underneath the nasal curtain, this could help to direct water flow through the nasal chamber as water is sucked into the mouth. The farther the nostrils are positioned from the mouth, the less likely the buccopharyngeal pump plays a meaningful role in nasal irrigation. Pressure differences between the outlet and inlet could also generate flow through the nose. If the incurrent and excurrent nostrils are positioned at right angles from each other, or at different heights, this could help stimulate flow into and through the nasal chamber. Specific odor harnessing mechanisms are more likely in some morphotypes than others.

The open nare morphotype, seen exclusively in guitarfishes and sawfishes, was easily identified and discriminated in all statistical tests. Batoids with the open nare morphotype are benthic or demersal and all BCF swimmers. This morphotype is fully exposed to the environment with visible lamellae, the absence of a nasal curtain, and a less distinct path for odor to follow for through the nasal chamber. The open nare morphotype was generally positioned close to the edge of the head and relatively far from the mouth. Because the open nare morphotype is not fully enclosed like the other morphotypes, it would be more difficult to generate a pressure difference between the inlet and outlet. Additionally, the buccopharyngeal pump may be less influential in odor capture in this morphotype.

The flush morphotype, the most common morphotype seen across a diversity of batoids, ranging from fast swimming, oscillatory eagle rays to slower swimming, undulatory round rays, was harder to statistically discriminate. While this morphotype has a diversity of inlet shapes, the flush morphotype may have the least specialized external morphology for odor capture. If a batoid is swimming parallel in the water column with flush nostrils on the underside of its head, odorants will have to make a sharp turn of up to 90° to enter the nasal chamber for sensory processing. This, coupled with the odor-impeding boundary layer, may make odor capture more difficult for the flush morphotype. However, this is not to say batoids with this nose type have reduced olfactory abilities. In fact, we know that eagle rays have some of the most sensitive noses (Schluessel et al. 2010). However, eagle rays are more pelagic, swim at faster speeds, and have their nostrils positioned more anteriorly on their head, which may make odor capture easier. Additionally, it is also possible that more specialized external morphologies did not provide a selective advantage for some species.

The protruding nare morphotype is seen across the batoid phylogeny and may represent convergence on an odor harnessing morphotype. The protruding nare morphotype may be an adaptation to overcome the challenge of the boundary layer, by projecting the incurrent nostril out of the boundary layer to make odor harnessing easier. The protruding nare morphotype is seen in batoids that are generally slower swimmers that associate closely with the ground, often in the deep sea, and display a “true” punting behavior (skates and electrics rays).

This study provides the first crucial step in better understanding batoid olfaction, by understanding the diversity of the morphology of the system. My analyses reveal that batoid nasal morphology is not just the result of shared ancestry but convergence on specific morphotypes. Specifically, the swimming mode of the animal was found to be most significant ecological factor. Because odor capture is a strictly hydrodynamic process, factors relating more directly to the fluid dynamics (i.e., swimming mode, velocity, Reynolds number) may be more important than other ecological factors in shaping the evolution of the diversity of batoid noses. Future studies should explore the fluid dynamics of odor capture of each morphotype, to determine if there are functional differences in the odor-harnessing mechanisms of these nose shapes.

## Supplementary Material

obac043_Supplemental_File

## Data Availability

The data collected for this study are available in the supplemental materials.

## Dedication

This paper is dedicated to the author's Masters advisor and Ph.D. coadvisor, Dr. Donald Buth, who recently passed away. The author recognizes his support and contribution to her academic success. She is grateful for the impact he had on her career.

## References

[bib1] Abel R L , Maclaine J S, Cotton R, Xuan V B, Nickels T B, Clark T H, Wang Z, Cox J P L. 2010. Functional morphology of the nasal region of a hammerhead shark. Comp Biochem Physiol A: Mol Integr Physiol 155:464–75. 19883784 10.1016/j.cbpa.2009.10.029

[bib2] Allen G R , Robertson D R. 1994. Fishes of the Tropical Eastern Pacific. Honolulu (HI): University of Hawaii Press. p. 34.

[bib3] Arkhipkin A I , Baumgartner N, Brickle P, Laptikhovsky V V, Pompert J H W, Shcherbich Z N. 2008. Biology of the skates *Bathyraja brachyurops* and *B. griseocauda* in waters around the Falkland Islands, Southwest Atlantic. ICES J Mar Sci 65:560–70.

[bib4] Aschliman N C , Nishida M, Miya M, Inoue J G, Rosana K M, Naylor G J. 2012. Body plan convergence in the evolution of skates and rays (*Chondrichthyes: Batoidea*). Mol Phylogenet Evol 63:28–42. 22209858 10.1016/j.ympev.2011.12.012

[bib5] Atema J . 1971. Structures and functions of the sense of taste in the catfish (*Ictalurus natalis*). Brain Behav Evol 4:273–94. 5118142 10.1159/000125438

[bib6] Bardach J E , Todd J H, Crickmer R. 1967. Orientation by taste in fish of the genus Ictalurus. Sci 155:1276–8. 10.1126/science.155.3767.1276 6018651

[bib7] Breder C M . 1926. The locomotion of fishes. Zoolo 50:159–297.

[bib8] Burns M D , Sidlauskas B L. 2019. Ancient and contingent body shape diversification in a hyperdiverse continental fish radiation. Evol 73:569–87. 10.1111/evo.13658 30560991

[bib8a] Cadena F , Jiménez I. 2018. Issues and Perspectives in Species Delimitation using Phenotypic Data: Atlantean Evolution in Darwin's Finches. Syst Biol 67:81–194. 10.1093/sysbio/syx071 28945876

[bib9] Camilieri-Asch V , Yopak K E, Rea A, Mitchell J D, Partridge J C, Collin S P. 2020. Convergence of olfactory inputs within the central nervous system of a cartilaginous and a bony fish: an anatomical indicator of olfactory sensitivity. Brain Behav Evol 95:139–61. 33171468 10.1159/000510688

[bib10] Collin S P , Kempster R M, Yopak K E. 2015. How elasmobranchs sense their environment. Fish Physiol 34:19–99.

[bib11] Collin S . 2012. The neuroecology of cartilaginous fishes: sensory strategies for survival. Brain Behav Evol 80:80–96. 22986825 10.1159/000339870

[bib12] Compagno L J V . 1999. Systematics and body form. In: Hamlet, W.C. (Ed.), Sharks, Skates, and Rays: The Biology of Elasmobranch Fishes. Baltimore (MD): Johns Hopkins University Press, p. 1–42.

[bib13] Cox J P L . 2008. Hydrodynamic aspects of fish olfaction. J R Soc Interface 5:575–93. 18184629 10.1098/rsif.2007.1281 PMC2408354

[bib14] Cox J P L . 2013. Ciliary function in the olfactory organs of sharks and rays. Fish and Fisheries 14:364–90.

[bib15] Eschmeyer W N , Herald E S, Hammann H. 1983. A field guide to Pacific coast fishes of North America. Boston (MA): Houghton Mifflin Company.

[bib16] Ferrando S , Gallus L, Ghigliotti L, Amaroli A, Abbas G, Vacchi M. 2017. Clarification of the terminology of the olfactory lamellae in chondrichthyes. Anat Rec 300:2039–45. 10.1002/ar.23632 28681530

[bib17] Froese, R. and Pauly D. Editors. 2022. FishBase. World Wide Web electronic publication. www.fishbase.org. (02/2022).

[bib17a] Gardiner J M , Atema J. 2010. The function of bilateral odor arrival time differences in olfactory orientation of sharks. Curr Biol 20:1187–91. 20541411 10.1016/j.cub.2010.04.053

[bib18] Gardiner J M , Hueter R M, Maruska K P, Sisneros J A, Casper B M, Mann D A, Demski L S. 2012. Sensory physiology and behavior of elasmobranchs (Ch 12). In: Carrier EC, Musick JA, Heithaus MR, editors. Biology of Sharks and their Relatives, 2nd Ed. (Boca Raton, FL: CRC Press).

[bib19] Gardiner J M , Atema J, Hueter R E, Motta P J. 2014. Multisensory integration and behavioral plasticity in sharks from different ecological niches. PLoS One 9:e93036. 24695492 10.1371/journal.pone.0093036 PMC3973673

[bib20] Garwood R J , Behnsen J, Haysom H K, Hunt J N, Dalby L J, Quilter S K, Maclaine J S, Cox J P. 2019. Olfactory flow in the sturgeon is externally driven. Comp Biochem Physiol A: Mol Integr Physiol 235:211–25 31229600 10.1016/j.cbpa.2019.06.013

[bib21] Garwood R J , Behnsen J, Ramsey A T, Haysom H K, Dalby L J, Quilter S K, Maclaine J S, Wang Z, Cox J P L. 2020. The functional anatomy of the pike, *Esox lucius* (L.). Comp Biochem Physiol A: Mol Integr Physiol 244:110688. 32171799 10.1016/j.cbpa.2020.110688

[bib22] Hara T J . 1993. Role of olfaction in fish behavior. In: Pitcher, TJ, editor. Behavior of Teleost Fishes, 2nd Ed. London: Chapman and Hall. p. 171–99.

[bib23] Hart N S , Collin S P. 2015. Shark senses and shark repellents. Integ Zool 10:38–64. 10.1111/1749-4877.12095 24919643

[bib24] Hart N S , Lisney T J, Collin S P. 2006. Visual communication in elasmobranchs. In: Ladich F, Collin SP, Moller P, Kapoor, BG, editors. Communication in Fishes. Enfield (NH): Science Publishers. p. 337–92.

[bib25] Jacobs L F . 2012. From chemotaxis to the cognitive map: the function of olfaction. Proc Natl Acad Sci 109:10693–700. 22723365 10.1073/pnas.1201880109 PMC3386877

[bib26] Johnsen P B , Teeter J H. 1985. Behavioral responses of bonnethead sharks (*Sphyrna tiburo*) to controlled olfactory stimulation. Marine Behav Physiol 11:283–91.

[bib27] Kass R E , Raftery A E. 1995. Bayes Factors. J Am Statist Assoc 90:773–95.

[bib28] Kajiura S M . 2001. Head morphology and electrosensory pore distribution of carcharhinid and sphyrnid sharks. Environ Biol Fishes 61:125–33.

[bib29] Kempster R M , McCarthy I D, Collin S P. 2012. Phylogenetic and ecological factors influencing the number and distribution of electroreceptors in elasmobranchs. J Fish Biol 80:2055–88. 22497416 10.1111/j.1095-8649.2011.03214.x

[bib30] Koester D M , Spirito C P. 2003. Punting: An unusual mode of locomotion in the little skate, *Leucoraja erinacea* (Chondrichthyes: Rajidae). Copeia 2003:553–61.

[bib31] Last, P. A., White W., de Carvalho M.R., Séret B., Stehmann M. and Naylor G.P. (Eds.). 2016a. Rays of the World. Melbourne: CSIRO Publishing.

[bib32] Last P R , Seret B, Naylor G J P. 2016b. *Rhinobatos borneensis* sp. nov. with a redefinition of the Rhinopristiformes. Zootaxa 4117:451–75. 27395187 10.11646/zootaxa.4117.4.1

[bib33] Lisney T J , Collin S P. 2007. Relative eye size in elasmobranchs. Brain Behav Evol 69:266–79. 17314474 10.1159/000100036

[bib33a] Lisney T J , Theiss S M, Collin S P, Hart N S. 2012. Vision in elasmobranchs and their relatives: 21st century advances. J Fish Biol 80:2024–54. 22497415 10.1111/j.1095-8649.2012.03253.x

[bib34] Macesic L J , Kajiura S M. 2010. Comparative punting kinematics and pelvic fin musculature of benthic batoids. J Morphol 271:1219–28. 20623523 10.1002/jmor.10865

[bib35] Mathewson R F , Hodgson E S. 1972. Klinotaxis and rheotaxis in orientation of sharks toward chemical stimuli. Comp Biochem Physiol Part A: Physiol 42:79–84. 10.1016/0300-9629(72)90369-6 4402725

[bib36] Meng Q , Yin M. 1981. A study of the olfactory organ of the sharks. Trans Chinese Ichthyol Soc 2:1–24.

[bib37] Meredith T L , Kajiura S M. 2010. Olfactory morphology and physiology of elasmobranchs. J Exp Biol 213:3449–56. 20889825 10.1242/jeb.045849

[bib38] Meredith T L , Caprio J, Kajiura S M. 2012. Sensitivity and specificity of the olfactory epithelia of two elasmobranch species to bile salts. J Exp Biol 215:2660–7. 22786643 10.1242/jeb.066241

[bib39] Michael S W . 1993. Reef sharks and rays of the world. A guide to their identification, behaviour, and ecology. vi, 107p. Monterey, California: Sea Challengers. J Marine Biol Assoc United Kingdom 73:987.

[bib40] Northcutt R G . 1978. Brain organization in the cartilaginous fishes. In: ES Hodgson, R F Mathewson. editors. Sensory biology of sharks, skates, and rays. Washington, D.C.: Office of Naval Research, Department of the Navy; p. 117–93.

[bib41] R Core Team . A language and environment for statistical computing. Vienna: R Foundation for Statistical Computing; 2019. Available from: https://www.r-project.org/.

[bib42] Rosenberger L J . 2001. Pectoral fin locomotion in batoid fishes: undulation versus oscillation. J Exp Biol 204:379–94. 11136623 10.1242/jeb.204.2.379

[bib43] Rosenberger L J , Westneat M W. 1999. Functional morphology of undulatory pectoral fin locomotion in the stingray *Taeniura lymma* (Chondrichthyes: Dasyatidae). J Exp Biol 202:3523–39. 10574730 10.1242/jeb.202.24.3523

[bib44] Rutledge K M , Summers A P, Kolmann M A. 2019. Killing them softly: ontogeny of jaw mechanics and stiffness in mollusk feeding freshwater stingrays. J Morphol 280: 796–808. 30950541 10.1002/jmor.20984

[bib45] Schaefer J T , Summers A P. 2005. Batoid wing skeletal structure: novel morphologies, mechanical implications, and phylogenetic patterns. J Morphol 264:298–313 15838841 10.1002/jmor.10331

[bib46] Schluessel V , Bennett M B, Bleckmann H, Blomberg S, Collin S P. 2008. Morphometric and ultrastructural comparison of the olfactory system in elasmobranchs: the significance of structure–function relationships based on phylogeny and ecology. J Morphol 269:1365–86. 18777568 10.1002/jmor.10661

[bib47] Schluessel V , Bennett M B, Bleckman H, Collin S P. 2010. The role of olfaction throughout juvenile development: functional adaptations in elasmobranchs. J Morphol 271:451–61. 19941378 10.1002/jmor.10809

[bib48] Settles G S . 2005. Sniffers: fluid-dynamic sampling for olfactory trace detection in nature and homeland security. J Fluids Eng 127:189–218.

[bib49] Simonitis L E , Marshall C D. 2022. Microstructure of the Bonnethead Shark (*Sphyrna tiburo*) Olfactory Rosette. Integ Organismal Biol 4:1. 10.1093/iob/obac027 PMC9293747 35860459

[bib50] Stein R W , Mull C G, Kuhn T S, Aschliman N C, Davidson L N, Joy J B. … Mooers A O. 2018. Global priorities for conserving the evolutionary history of sharks, rays and chimaeras. Nat Ecol Evol 2:288–98. 29348644 10.1038/s41559-017-0448-4

[bib51] Takami S , Luer C A, Graziadei P P C. 1994. Microscopic structure of the olfactory organ of the clearnose skate, *Raja eglanteria*. Ital J Anat Embryol 190:211–30. 10.1007/BF00234300 7818093

[bib52] Tester A L . 1963. Olfaction, gustation, and the common chemical sense in sharks. In: Gilbert, P.W. (Ed.), Sharks and Survival. D.C, Heath and Co, Lexington, pp. 255–82.

[bib53] Theiss S M , Hart N S, Collin S P. 2009. Morphological indicators of olfactory capability in wobbegong sharks (*Orectolobidae, Elasmobranchii*). Brain Behav Evol 73:91–101. 19321959 10.1159/000209865

[bib54] Theisen B , Zeiske E, Breucker H. 1986. Functional morphology of the olfactory organs in the spiny dogfish (*Squalus acanthias*) and the small spotted catshark (*Schliorhinus canicula*). Acta Zoologica 67:73–86.

[bib55] Timm L L , Fish F E. 2012. A comparative morphological study of the head shape and olfactory cavities of sharks inhabiting benthic and coastal/pelagic environments. J Exp Mar Biol Ecol 414–415:75–84.

[bib56] Uyeda J C , Caetano D S, Pennell M W. 2015. Comparative analysis of principal components can be misleading. Syst Biol 64:677–89. 25841167 10.1093/sysbio/syv019

[bib57] Vogel S . 1977. Flows in organisms induced by movements of the external medium. In Scale effects in animal locomotion (ed. Pedley T J), pp. 285–97. London, UK: Academic Press.

[bib58] Webb P W . 1998. Swimming. In The Physiology of Fishes, second edition (ed. D. H. Evans), pp. 3–24. New York: CRC Press.

[bib59] Wilga C A , Lauder G V. 2002. Biomechanics of locomotion in sharks, rays, and chimeras. Biology of sharks and their relatives, 5:139–164.

[bib60] Yopak K E , Lisney T J, Collin S P. 2015. Not all sharks are “swimming noses:” variation in olfactory bulb size in cartilaginous fishes. Brain Structure and Function 220(2):1127–43. 24435575 10.1007/s00429-014-0705-0

[bib61] Yopak K E , McMeans B C, Mull C G, Feindel K W, Kovacs K M, Lydersen C, Fisk A T, Collin S P. 2019. Comparative brain morphology of the Greenland and Pacific Sleeper Sharks and its functional implications. Sci Rep 9:10022. 31296954 10.1038/s41598-019-46225-5 PMC6624305

[bib62] Zeiske E , Theisen B, Gruber S H. 1987. Functional morphology of the olfactory organ of two carcharhinid shark species. Can J Zool 65:2406–12.

